# Supplementary material for: Methods for dealing with unequal cluster sizes in cluster randomized trials: A scoping review
Source: PLoS One. 2021 Jul 29;16(7):e0255389. doi: 10.1371/journal.pone.0255389 (PMC8320970; doi:10.1371/journal.pone.0255389)
Supplement: S1 Appendix — (DOCX) [file pone.0255389.s001.docx]

**S1 Appendix:**

**The search steps in Medline and EMBASE:**

1. (stepped wedge or stepped-wedge).ab,kf,ti.

2. (Network-randomi?ed or network randomi?ed).ab,kf,ti.

3.(pseudocluster randomi?ed or pseudo cluster randomi?ed) or pseudo-cluster randomi?ed.ab,kf,ti.

4. group treatment.ab,kf,ti.

5. (group$ adj2 randomi$).ab,kf,ti.

6. (cluster$ adj2 randomi$).ab,kf,ti.

7. (community adj2 randomi$).ab,kf,ti.

8. unequal cluster size$.ab,kf,ti.

9. (imbalanc$ adj2 cluster$).ab,kf,ti.

10. sample size$.ab,kf,ti.

11. Cluster size$.ab,kf,ti.

12. (power or efficiency).ab,kf,ti.

13. (Random effects or mixed effects or hierarchical or generaliz$ estimat$ equation$ or multi-level or multi level or multilevel or cluster level).ab,kf,ti.

14. (ICC or intracluster correlation or intraclass correlation).ab,kf,ti.

15. (intra-cluster correlation or intra-class correlation).ab,kf,ti.

16. variance inflation factor.ab,kf,ti.

17. (coefficient of variation or coefficient of variance).ab,kf,ti.

18. optimal design$.ab,kf,ti.

19. optimized design$.ab,kf,ti.

20. restricted randomi$.ab,kf,ti.

21. constrained randomi$.ab,kf,ti.

22. covariate balance.ab,kf,ti.

23. covariate imbalance.ab,kf,ti.

24. bias.ab,kf,ti.

25. 1 or 2 or 3 or 4 or 5 or 6 or 7

26. 8 or 9 or 10 or 11 or 12 or 14 or 15 or 16 or 17 or 18 or 19 or 20 or 21 or 22 or 23

27. 10 or 11

28. (type adj2 error).ab,kf,ti.

29. 26 or 28

30. 25 and 29

31. 12 or 24 or 28

32. 13 and 27 and 31

33. 30 or 32

34. remove duplicates from 30

35. remove duplicates from 32

36. 34 or 35

37. remove duplicates from 36
