# Supplementary material for: Methods for dealing with unequal cluster sizes in cluster randomized trials: A scoping review
Source: PLoS One. 2021 Jul 29;16(7):e0255389. doi: 10.1371/journal.pone.0255389 (PMC8320970; doi:10.1371/journal.pone.0255389)
Supplement: Attachment — Submitted filename: 2021.3.21-response to reviewers.docx [file pone.0255389.s004.docx]

**Response to Editor’s and Reviewers’ Comments**

- **Title of Manuscript:** Methods for dealing with unequal cluster sizes in cluster randomized trials: A scoping review
- **Authors:** Denghuang Zhan, Liang Xu, Yongdong Ouyang, Richard Sawatzky, Hubert Wong
- **Affiliation:** School of Population & Public Health, University of British Columbia, Vancouver, BC, Canada; Centre for Health Evaluation and Outcomes Sciences, St Paul’s Hospital, University of British Columbia, Vancouver, BC, Canada; School of Nursing, Trinity Western University, Langley City, British Columbia, Canada.
- **Manuscript ID:** PONE-D-20-23827

We would like to thank the editor for coordinating the review process for this submission. We would also like to thank the reviewers for their constructive comments which helped us improve our work. Point-to-point responses to the reviewers’ comments are given below.

**Reviewer #1**: The manuscript focuses on recent research studies on cluster randomized trials (CRTs) with unequal cluster sizes. The authors searched and studied the related papers from several literature databases and the inclusion steps are also presented in detail. Among the 67 papers included, the authors discussed the impacts of unequal cluster sizes on both design and analysis of cluster randomized trials by trial type. The manuscript provides a valuable resource on dealing with unequal cluster sizes for researchers working on different types of cluster randomized trials.

**Major comments:**

***1.1*** *The authors gave a broad review on CRTs by trial type and analyzed the impact of unequal cluster sizes on both type I error and power calculation on a case-by-case basis. Each specific case, however, does no form natural transitions in terms of methods or CRT parameters. For example, the subsection of continuous outcomes in section 3.1.1 on page 10 has summarized several papers related to analysis considerations on continuous outcomes in parallel-arm CRTs with unequal cluster sizes. However, the settings, implications or conclusions vary by paper and there are no transition sentences connecting the different papers. Therefore, in addition to the similar case in the same subsection, I think grouping the papers within each subsection to describe might give more clear information and the grouping could be based on methods utilized or similar scopes. In addition, could the authors add a summarizing sentence at the beginning of each subsection to guide the readers on the specific impact of unequal cluster sizes to that subsection before describing different papers one-by-one?*

**Thank you for these comments. We have re-organized the structure into subsections based on the combination of type of design (p-CRT, SW, etc) and type of outcome (Gaussian, binary, etc) and discuss within each subsection both design and analysis concerns. This should enable a reader to more easily identify the information that would be relevant to their trial (as type of design and type of outcome are usually pre-selected). In addition, the Supporting Information paper information table has been re-organized into multiple tables to match this structure and enable readers to quickly see the scope of work investigated in each paper.**

**We have added summary sentences when there appears to be a clear, general conclusion. However, in most sections, the magnitude of impact of unequal cluster sizes varies substantially with changes in other input parameters (# of clusters, cluster size CV, ICC, etc) so we believe it would be more prudent to guide readers to specific papers rather than make definitive judgements based on somewhat arbitrary judgments/cutoffs (e.g., a claim such as ‘unequal cluster sizes should not be ignored if the CV is greater than 0.23’, except where we are simply reporting what other authors have suggested).**

***1.2*** *Could the authors give slightly more information when mentioning certain number of methods? I found two: 1) on page 17, “Breukelen et al. [55] also compared three RE equations and claimed that the efficiency loss due to varying …”; 2) on page 21, “They compared empirical power obtained using the DE derived by Woertman et al. [75] and the two proposed adjusted DEs…”. Could the authors indicate the general techniques which the three and two methods are based on?*

**We have added details as suggested: “Breukelen et al. [41] also compared one RE equation proposed by Manatunga et al. [23] and two RE equations proposed by Breukelen et al. [35] together and claimed that the efficiency… ” and “The two adjusted DEs are derived based on the cluster weights and minimum variance weights, respectively. They compared empirical power obtained…”**

**Minor comments:**

***1.3*** *Table 1 summarizes the methods, trial types and scopes of each paper. Could the authors also give some summary statistics of the parameters considered in the manuscript for different types of CRTs? On page 8, we have the overall ranges for some parameters such as number of clusters and it might bring more comprehensive information about parameters utilized by type for readers.*

**Table 1 is intended to provide a “birds-eye” view of the available literature and we believe adding details about parameter ranges investigated to the table would make it difficult to read. However, our (new) Supporting Information tables have been re-organized based on the combination of type of design (p-CRT, SW, etc) and type of outcome (Gaussian, binary, etc) to enable readers to quickly identify the papers that cover parameter ranges that could be relevant to their specific trial.**

**Reviewer #2:** The manuscript concerns the scoping review of the literature on statistical methods in cluster randomized trial with unequal cluster sizes. By searching through EMBASE, Medline, Google Scholar, Web of Science, and MathSciNet, the authors identified relevant papers and summarized the papers in terms of trial design, outcome measure, model, and statistical ob- jectives on analysis, simulation, relative efficiency corrections, and design effect. They found that methods for the p-CRT with a Gaussian outcome with unequal cluster sizes in have been investigated quite broadly, but limited investigations have been done outside of the p-CRT with Gaussian outcome.

*The main body on the results of the scoping review is in Section 3, where the authors concerns different designs in different sub sections. It seems to me this section is more like a listing of each paper described by one sentence, on what they did, rather than a “review” with some summarization and comparison.*

**Thank you for this comment. We have added summary sentences when there appears to be a clear, general conclusion. However, please see our response to Reviewer #1, comment 1.1 for our explanation of why this is not always achievable.**

***2.1*** *The methods on CRTs with unequal cluster sizes were categorized to analysis (Type 1 error, bias, coverage) and design (power and size calculation), which is further described by simulations, relative efficiency corrections and design effect and related corrections, as shown in Table 1. I’m quite confused about those categories, and there seems not descriptions on how those categories are defined. In my understanding, analysis is to obtain estimate of parameter of interest related to data collected from CRTs, and design is on obtaining cluster sizes to attain desired power. Therefore, (1) I believe type 1 error is indeed a measure to evaluate study design, such that it shall be in the category of “design”; (2) simulations are always conducted to evaluate performance, in both analysis and design approaches, so I don’t understand why it shows up in “design” category parallel to RE and DE corrections.*

**Thank you for pointing out the ambiguity in the use of the term “simulation”. As the reviewer points out, it could be used to describe the methodology for evaluating the performance of a particular method, or simply to describe the computational tool for obtaining, say, the power (because the analytic calculation is intractable). The latter meaning is the intended one in this table and the column heading has been changed to “Sample size/power via simulation”.**

**We agree with the reviewer that type 1 error is relevant to study design since all power calculations are underpinned by an assumed analysis method. However, we feel that nonetheless, the statistical issues concerning analysis methods exist independent of design considerations and can be discussed separately.**

***2.2*** *The main searching criterion is shown in Fig 1, about which I have a few questions. The right oval in Branch A for design methodology includes key words related to unequal cluster sizes or those relevant to trial design, e.g., sample size. Therefore, based on this criterion, I would imagine a large number of papers that do not ever consider unequal cluster sizes would be included, since a very general key word (e.g., sample size) is used. I wonder why the searching criterion is set up as so. In addition, in the introduction, the authors mentioned other names for CRTs, for example place-based trials. Would this current searching criterion miss the papers with “place-based trials” indicating CRTs with unequal cluster sizes?*

**The reviewer is correct that a large number of hits was generated using general key words such as “sample size”. Our concern was that some methodological papers may investigated unequal cluster sizes as a small component of larger study, so may not have included any reference to unequal cluster sizes in their key words. Using more general key words allowed us to catch such papers for review of this possibility. We have updated our search criterion to include additional term “cluster level” in category 5 in Branch B and extended the end date of the search to find the most recently published papers- we identified another 12 papers since April 2020.**

***2.3*** *For Branch B for analysis methodology, the found paper would always include “random effects” or “mixed effects” or “hierarchical” or “‘generalized estimating equations” or “multi- level”, as indicated by Fig 1. I understand those are common methods to address clustered trials, however, this automatically restricts the analysis methods, and any other analysis methods outside this range cannot be found.*

**Thank you for pointing out this limitation. We agree that the specific terms of the analysis methodology would limit the search. However, our search results showed that the papers failed to be captured by Branch B could still be captured by Branch A. We have added more terms such as “cluster level” in category 5 in Branch B, please see response to Comment 2.2.**

Here are some other comments:

***2.4*** *The review of papers are categorized by trial designs, i.e., p-CRT, sw-CRT, pn-CRT, and co-CRT. Even though those designs were first mentioned in the introduction, and the statistical methods for different trial designs with equal cluster sizes were discussed then, the definitions were given in Section 3. It would potentially cause trouble for readers not familiar to different trial designs. Please consider defining those designs in the introduction.*

**We agree that it would be more natural to provide descriptions of the design types in the introduction and indeed that was done in early drafts. However, we felt this led to the introduction taking too long to get to the key motivations for and the aims of the review, so we feel deferring the presentation of these descriptions to later improves the readability, but we would be happy to defer to the judgment of the AE and the reviewers.**

***2.5*** *Page 3, line 68: “Except within a very restricted set of conditions (e.g., with multivariate normal outcomes and equal cluster sizes), exact formulae for obtaining desired statistical properties (e.g., bias, Type I error rates, power, etc.) are not available and most formulae have been derived under the assumption that the number of clusters and the sample sizes within clusters are sufficiently large for results from asymptotic theory to hold”. This paragraph is on existing reviews on statistical methods of CRTs with equal sizes. Not sure how this sentence on statistical properties depending on sufficiently large cluster numbers and sizes is relevant.*

**Thank you for pointing the absence of continuity here. We have moved this sentence to the following paragraph and modified it (along with a sentence there) as follows:**

**“Exact formulae for obtaining desired statistical properties (such as bias, Type I error rates, power, etc.) are available only under very restricted conditions (e.g., with multivariate normal outcomes with equal cluster sizes), and typically, asymptotic approximations must be used. However, when cluster sizes are unequal and potentially small, calculations based on these asymptotic approximations may be highly inaccurate and this issue should be considered during both the trial design as well as the data analysis stages.”**

***2.6*** *One of the search engines, MathSciNet, stated in Section 2.1 is not mentioned in the Abstract.*

**Thank you, we added this search engine in the abstract.**

***Reviewer #3***: Line 20: This could explicitly state that it is the number of participants enrolled that might vary across clusters, to make it clear it is not referring to natural cluster size

**Edited as suggested. Thank you.**

Line 22: very minor point but saying CRT design could be interpreted as literature on the design of crt’s, which is not the case here as the authors are looking at design and analysis literature. See also line 66

**Thank you for this observation. In fact, this wording was a conscious choice on our part as we felt that it could be mis-leading to suggest that most *analysis* literature has assumed equal cluster sizes!**

line 37, while the authors have identified a gap in the methodological literature for alternative outcomes it would be helpful to say something about whether, from the type of trials actually being conducted, there is a need to fill this gap

**Thank you for this observation. We have added the following clause:**

**“…, particularly methodology involving binary outcomes, the most commonly used type of primary outcome in trials.”**

**Also, we would argue that although other types of outcomes may not be used commonly, the best time to develop methodology is before it is needed (a likely opine from any statistician who has spent sleepless nights accommodating requests for a novel design right before a grant deadline).**

line 46: after the first mention of group (cluster) could the terminology of cluster be consistently used as this is the most commonly used terminology.

**Edited as suggested. Thank you.**

Line 58. The authors introduce common methods of analysis here, it could be useful to similarly introduce sample size strategies: de, re, that are later mentioned in table 1

**Thank you for this suggestion. We have added the following sentence in the Introduction:**

**“Strategies to correct sample size calculations for unequal cluster sizes include evaluating relative efficiencies or design effects based on the cluster size CV.”**

Line 63: the order of references 5,6 need to be switched in order for this sentence to be accurate.

**Corrected. Thank you for catching that.**

Line 62: It would be helpful to describe the context or drawbacks of the 2 Turner reviews which necessitate the need for this scoping review. Were these similarly confined to p-crt? This paragraph might sit better near the end of this section to lead into the justification and scope of this review

**Thank you for this suggestion. We have added the following sentence in the final paragraph of the introduction:**

**“Previous literature reviews have identified various key papers (e.g., 11 papers in Turner et al. [5], 8 papers in Turner et al. [6]), but no comprehensive review appears to have been published.”**

Line 73: Could the authors provide evidence of this statement as well as the frequency of trials with low numbers of clusters.

**We have added three references to support this statement.**

**(https://academic.oup.com/ije/article/47/1/321/4091562#114554644;**

**https://link.springer.com/article/10.1186/s13063-016-1571-2;**

**https://bmjopen.bmj.com/content/bmjopen/7/11/e017151.full.pdf)**

Line 87: would be helpful to define the coefficient of variation in cluster size here for those unfamiliar

**We have edited the sentence as follows: “… when the coefficient of variation (CV) of the cluster sizes, defined as the ratio of the standard deviation of the cluster sizes to the mean cluster size …”**

Line 106: is studies the appropriate word to describe these papers

**Thank you. We have changed the word “studies” to “papers”.**

Fig 1: was any consideration given to randomisation spelt with an s rather than z.

**Thank you for catching this. We actually used both “s” and “z” in our search (as shown in S1 Appendix). We corrected it in the Fig 1.**

Line 211 is this the CV of cluster size or CV as alternative to ICC

**It was the cluster size CV, but this sentence no longer appears in the revised manuscript.**

Section 3.1.1 it would be helpful to include more of a summary here, particularly what the gaps were before delving into the details for the different outcomes.

Line 218 +: The organisation of these sections need to be improved. As a reader I would likely know the CV and ICC, number of clusters in my study and I would want to be able to quickly identify the methods that have been tested in similar conditions. This information gets somewhat lost in the text making it harder to read. Perhaps subheadings for the CV and then the ICC and cluster sizes presented at the beginning of each paragraph would help. Alternatively a summary for each analysis method (or the most common/easily implemented) and the conditions under which it works best rather than describing each paper in turn. The authors touch upon the lack of synthesis of findings in their discussion but its not clear why no synthesis has been done here as I think that is where value can be added.

**Thank you for these suggestions. We have re-organized the materials to make it easier for readers to quickly identify the papers/results that could be relevant to their specific trial. Specifically, readers can now skip to the subsection for a specific combination of trial design + outcome type. Then, for that combination, supplementary tables have been provided identifying the range of trial parameter values (CV, ICC, etc) that each paper investigated.**

**Our aim in this paper is to assist readers in identifying literature that could be relevant to their application. We fully agree that a synthesis with recommendations on best methodological choices would be very valuable, but please see our response to Reviewer #1, comment 1.1 for our explanation of why this is not achievable.**

Section 3.1.2 Given there are several reviews that have already considered these methods for the parallel group study is this section necessary or could it be reduced to those methods that had not been identified in previous reviews?

**Thank you for raising this point. While we acknowledge that our paper may replicate some materials from previous reviews, we believe listing all of the papers here adds value by making it easier for readers to find what is relevant. We have revised this section for continuous and binary outcomes separately and hopefully it will be easier for readers to follow.**
