# Supplementary material for: Methods for dealing with unequal cluster sizes in cluster randomized trials: A scoping review
Source: PLoS One. 2021 Jul 29;16(7):e0255389. doi: 10.1371/journal.pone.0255389 (PMC8320970; doi:10.1371/journal.pone.0255389)
Supplement: Attachment — Submitted filename: 617response_unequal cluster size scoping.docx [file pone.0255389.s005.docx]

**Response to Editor’s and Reviewers’ Comments**

- **Title of Manuscript:** Methods for dealing with unequal cluster sizes in cluster randomized trials: A scoping review
- **Authors:** Denghuang Zhan, Liang Xu, Yongdong Ouyang, Richard Sawatzky, Hubert Wong
- **Affiliation:** School of Population & Public Health, University of British Columbia, Vancouver, BC, Canada; Centre for Health Evaluation and Outcomes Sciences, St Paul’s Hospital, University of British Columbia, Vancouver, BC, Canada; School of Nursing, Trinity Western University, Langley City, British Columbia, Canada.
- **Manuscript ID:** PONE-D-20-23827

We would like to thank the editor for coordinating the review process for this submission. We would also like to thank the reviewer for the constructive comments which helped us improve our work. We have responded to the reviewer’s comments below.

**Reviewer #1**: *The authors have made substantial changes on the manuscript, which reviews recent studies on cluster randomized trials (CRTs) with unequal clusters. The revised manuscript now has a clearer structure with sections of trial type, outcome type and design/analysis aspect. The supporting information also provide detailed literature tables that align with the manuscript sections. These changes help readers find relevant material for their uses in CRTs. The authors have addressed my comments on elucidating several methods in the manuscript and also presenting more comprehensive statistics of parameters of each paper in the supporting information tables.*

*However, the revised manuscript still does not address my previous concern that there is a lack of connections when reviewing the recent papers. Rather than grouping and comparing papers, the authors seem to list and describe the papers that fall into the combination of specific trial type, outcome type and design/analysis aspect. I agree with Reviewer 2 that the review structure follows a one-by-one listing and summarizing process, instead of connecting or comparing the papers.*

***Major comments:***

*Although the authors added some summarizing sentences that gives general information on the quantity of papers falling into a category, I expect summaries on the papers described and the connections among them. I agree with the authors that there might not be a general conclusion of papers with very different settings, but the summaries and the connections/transitions can also be an introduction, a view or a comment. For example, in the analysis aspect of continuous outcomes of parallel-arm CRTs, the authors can describe the method trend (GEE, mixed models or others) in this part before going to each paper with its detail. In addition, they can also make more comments on the transitions of papers when they share similar methods, objectives, or conclusions. After all, why are the papers grouped in the same paragraph besides the fact that they fall into the same trial-outcome-design/analysis combination? Can their connections be presented and reviewed more smoothly?*

**Thank you for these comments. We have performed substantial reorganization of several sections to group papers that have overlap and smooth the presentation. When possible, we have made connections/comparisons between papers. This was done primarily on the analysis aspects as suggested by the reviewer – comparing performance of different methods for power calculations is problematic typically, as variation in power between methods is driven greatly by differences in the Type I error rates of the underlying analysis methods. We acknowledge that parts of the manuscript read as a “one-by-one listing”, but in these cases, it was difficult (or we judged it imprudent) to compare to other papers which superficially appeared to address the same questions in the same setting, but were in fact quite different on close examination (e.g., differing simultaneously in both the assumed analytic model and the ranges of input parameter values (# of clusters, cluster size CV, ICC, etc.), making it impossible to ascertain which aspect(s) explain differences in findings).**

**We re-iterate that this is a scoping review whose primary intent is to point readers to literature that may be useful to them, not a systematic review to evaluate the methods and advise readers on which ones ought to be used. Indeed, as a result of conducting the scoping review and seeing the many gaps that exist, we believe it would be difficult to achieve the latter aim. We believe, however, that this paper has achieved the former aim.**
